# Characterizing the metabolic heterogeneity in human breast cancer xenografts by 3D high resolution fluorescence imaging

**DOI:** 10.1186/2193-1801-2-73

**Published:** 2013-02-28

**Authors:** He N Xu, Gang Zheng, Julia Tchou, Shoko Nioka, Lin Z Li

**Affiliations:** 1 Molecular Imaging Laboratory, Department of Radiology, Perelman School of Medicine, University of Pennsylvania, Pennsylvania, USA; 2 Britton Chance Laboratory of Redox Imaging, Johnson Research Foundation, Department of Biochemistry and Biophysics, Perelman School of Medicine, University of Pennsylvania, Pennsylvania, USA; 3 Department of Medical Biophysics, University of Toronto, Toronto, Canada; 4 Department of Surgery, Perelman School of Medicine, University of Pennsylvania, Pennsylvania, USA; 5 Rena Rowan Breast Center, Abramson Cancer Center, Perelman School of Medicine, University of Pennsylvania, Pennsylvania, USA

**Keywords:** The Chance redox scanner, Redox scanning, Glucose uptake, NADH, Flavoprotein (Fp), FAD, Mitochondrial redox state, Pyro-2DG, MCF-7, MDA-MB-231, MDA-MB-468

## Abstract

We previously reported that tumor mitochondrial redox state and its heterogeneity distinguished between the aggressive and the indolent breast cancer xenografts, suggesting novel metabolic indices as biomarkers for predicting tumor metastatic potential. Additionally, we reported that the identified redox biomarkers successfully differentiated between the normal breast tissue and the cancerous breast tissue from breast cancer patients. The aim of the present study was to further characterize intratumor heterogeneity by its distribution of mitochondrial redox state and glucose uptake pattern in tumor xenografts and to further investigate the metabolic heterogeneity of the clinical biopsy samples. We employed the Chance redox scanner, a multi-section cryogenic fluorescence imager to simultaneously image the intratumor heterogeneity in the mitochondrial redox state and glucose uptake at a high spatial resolution (down to 50 × 50 × 20 μm^3^). The mitochondrial redox state was determined by the ratio of the intrinsic fluorescence signals from reduced nicotinamide adenine dinucleotide (NADH) and oxidized flavoproteins (Fp including FAD, i.e., flavin adenine dinucleotide), and the glucose uptake was measured using a near-infrared fluorescent glucose-analogue, pyropheophorbide 2-deoxyglucosamide (Pyro-2DG). Significant inter- and intratumor metabolic heterogeneity were observed from our imaging data on various types of breast cancer xenografts. The patterns and degrees of heterogeneity of mitochondrial redox state appeared to relate to tumor size and metastatic potential. The glucose uptake was also heterogeneous and generally higher in tumor peripheries. The oxidized and reduced regions mostly corresponded with the lower and the higher pyro-2DG uptake, respectively. However, there were some regions where the glucose uptake did not correlate with the redox indices. Pronounced glucose uptake and high NADH were observed in certain localized areas within the tumor necrotic regions, indicative of the existence of viable cells which was also supported by the H&E staining. Significant heterogeneity of the redox state indices was also observed in clinical specimens of breast cancer patients. As abnormal metabolism including the Warburg effect (high glycolysis) plays important roles in cancer transformation and progression, our observations that reveal the 3D intratumor metabolic heterogeneity as a characteristic feature of breast tumors are of great importance for understanding cancer biology and developing diagnostic and therapeutic methods.

## Introduction

Abnormal metabolism and high variability in disease presentations are among the hallmarks of cancer (Heppner & Miller 1998; Hanahan & Weinberg 2011). The intratumor heterogeneity or diversity has been associated with tumor progression and aggressiveness (Heppner 1984; Allred et al. 2008; Park et al. 2010). It is therefore of great importance to characterize tumor metabolic heterogeneity.

Cancer metabolism has been an active research area for understanding tumor biology and developing biomarkers for cancer diagnosis and monitoring treatment response (Cairns et al. 2011; Vander Heiden et al. 2009). Higher than normal glucose uptake/metabolism as first identified by Otto Warburg in 1920s (Koppenol et al. 2011), lays the foundation for cancer staging by fluorinated 2-deoxyglucose positron emission tomography (FDG-PET) in the clinic. Studies also investigated the glucose uptake and its spatial heterogeneity in animal models (Kallinowski et al. 1988; Dearling et al. 2004). Another area under active research investigation is mitochondrial metabolism including TCA cycle and oxidative phosphorylation. Several mitochondrial metabolic enzymes have been identified as oncogenes or tumor suppressors in some cancers (Thompson 2009).

Previously we reported that the mitochondrial redox state and its heterogeneity in tumors imaged by the Chance redox scanner provide sensitive and potentially diagnosis-useful indices for differentiating among five human melanoma lines and two breast cancer lines with different metastatic potential in mouse models (Li et al. 2009a; Xu et al. 2010) . In addition, we reported that the redox imaging indices differentiated between the normal and cancerous breast tissues from breast cancer patients (Xu et al. 2013a). We showed that the aggressive or metastatic tumors have localized more oxidized areas (higher Fp redox ratio, Fp/(Fp + NADH)), and that the Fp redox ratios of the oxidized areas positively correlated with tumor metastatic potential and thus could be further developed for grading tumor aggressiveness of human melanoma and breast cancer. The pattern of the oxidized areas with surrounding reduced areas appears to be a distinct feature of intratumor heterogeneity in aggressive tumors. However, the 3D heterogeneity of the mitochondrial redox indices (NADH, Fp, and the Fp redox ratio) has not been thoroughly investigated for whole tumors.

Because of cancer cells’ higher than normal glycolysis, simultaneously imaging the glucose uptake/metabolism and the mitochondrial redox state in tumors adds one more dimension to our understanding of tumor metabolism. As the size of an oxidized tumor area can be as small as 1–2 mm (Xu et al. 2010), FDG-PET is not suitable for such a purpose due to its low spatial resolution (1–2 mm for small animals and ~4 mm for human subjects). Previously, Zheng and co-workers developed the near-infrared fluorescent glucose-analog Pyro-2DG and also confirmed that Pyro-2DG was selectively accumulated in tumor cells via the glucose transporters (Zhang et al. 2003). Using the high-resolution Chance redox scanner, they achieved simultaneous imaging of Pyro-2DG uptake and the redox indices of the 9L glioma and c-MYC-induced mammary tumors in animal models (Zhang et al. 2004). The imaging data showed that Pyro-2DG uptake correlated well with the reduced redox state and did not affect the redox state of the tumor tissue.

In the present study, we first report the 3D distributions of the mitochondrial redox state in the three breast tumor xenografts in nude mice. We also report the significant intra-tumor heterogeneity of the mitochondrial redox state observed in tumor samples from breast cancer patients. We then show the glucose uptake distribution pattern and its correlations with the redox indices in the breast tumor xenografts. A portion of the preliminary data from mouse models were published in a conference proceeding (Xu et al. 2012; Xu et al.2011a). The results are useful for deepening our understanding on tumor metabolism and its heterogeneity, which may drive the development of novel diagnostic and therapeutic methods for cancer management.

## Materials and methods

### Tumor models and sample preparation for redox scanning

The animal protocols were approved by the Institutional Animal Care and Use Committee at the University of Pennsylvania. Subcutaneous xenografts of three human breast cancer cell lines with increasing metastatic potential (MCF-7 < MDA-MB-468 < MDA-MB-231, N ≥ 5 for each line) were grown to 6–10 mm in diameter in athymic nude mice for the 3D redox scanning. Another two MDA-MB-231 tumor xenografts (>10 mm in diameter and with an apparent necrotic center, average tumor volume = 989 mm^3^) and two MCF-7 tumor xenografts (>10 mm in diameter, average tumor volume = 750 mm^3^) were used for simultaneous imaging of Pyro-2DG uptake and the redox state. Pyro-2DG was administered to the anesthetized mice through tail vein injection at a dosage of ~2.5 mg/kg after the tumor-bearing mice were starved for 24 hrs. In approximately two hours, the anesthetized mice were snap-frozen using liquid nitrogen. The snap-freezing protocol maintained the *in vivo* mitochondrial redox state for the *ex vivo* redox scanning. The preparation of tissue samples for the redox scanning were previously described (Xu et al. 2010; Xu et al. 2009a). Briefly, the excised frozen samples were embedded with chilled mounting buffer (ethanol:glycerol:water = 10:30:60). Frozen reference standards of NADH, FAD, and Pyro-2DG with known concentrations (in 10 mM Tris–HCl buffer, pH ~7) were quickly mounted adjacent to the tissue for the purpose of calibrating the concentrations of these fluorescent molecules in tissue.

### Human breast tumor tissue collection

Tissue collection from patients was performed according to a protocol approved by the Internal Review Board of the University of Pennsylvania. Both normal and cancerous tissue specimens were collected from the cancer-bearing breasts of three patients shortly after surgical resection. Core biopsies and/or thin tissue blocks were obtained from tumor and normal adjacent breast tissue, respectively and were snap-frozen in liquid nitrogen within 5–15 minutes after the tissue removal from the body. These tissue specimens were embedded in the same way as described for the xenografts aforementioned.

### Redox scanning using the chance redox scanner

The low-temperature redox scanner developed by Chance et. al (Chance et al. 1979; Gu et al. 2002; Li et al. 2009b; Quistorff et al. 1985) (the Chance redox scanner) was employed to obtain the multi-slice fluorescence images of NADH, Fp, and Pyro-2DG. The optical filters were 430 nm ± 25 nm and 525 nm ± 32 nm for Fp excitation and emission channel respectively; 360 nm ± 13 nm and 430 nm ± 25 nm for NADH excitation and emission channel, respectively; and 430 ± 28 nm and 670± 11 nm for pyro-2DG excitation and emission, respectively. For the 3D imaging study, the tumor samples were scanned section by section at different tissue depths with 400 ~ 800 μm spacing. Most tumors were scanned at least to the center. For some tumors the entire tumor was scanned. For the study of simultaneous imaging of NADH, Fp, and pyro-2DG, the fluorescent signals were obtained from 2 ~ 3 tissue sections with depth ranging from ~1700-3000 μm under the mouse skin.

### Data analysis

The nominal concentration of NADH, Fp, and pyro-2DG in tissue was interpreted using the fluorescence intensity of the corresponding reference standard, respectively and further used to calculate Fp/(NADH + Fp) or NADH/(Fp + NADH). To account for the cell or mitochondria density difference at different regions within a tumor, the normalized Pyro-2DG, i.e. Pyro-2DG/(NADH + Fp) was also calculated based on the nominal concentrations of NADH and Fp.

In order to obtain the mean values in the more oxidized regions (high Fp redox ratio or Fp/(NADH + Fp)) and less oxidized regions (low Fp redox ratio), the sum of two Gaussian functions  was fitted to the histograms of the NADH, Fp, and Fp redox ratio images (Xu et al.2013b). The fitted b_i_ (i = 1, 2) values were taken as the means of these redox indices in the corresponding more or less oxidized region.

## Results

### 3D redox images show high degree intertumor and intratumor heterogeneity

**Figure 1:**
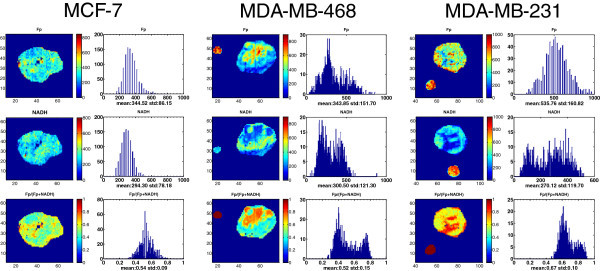
**Typical images of redox scanning of the mouse xenografts of MCF-7 (left, depth d = 3400 μm), MDA-MB-468 (middle d = 1200 μm), and MDA-MB-231 (right, d = 800 μm) tumor lines.** The Fp or NADH redox ratio ranges between 0 and 1; the Fp or NADH images are in the unit of μM in reference to the corresponding standards. The x axes of the corresponding histograms represent the Fp redox ratio or concentration. The y axes represent the number of pixels in the tumor section having a specific value of Fp redox ratio or Fp and NADH concentration. The small round spots outside the tumor section are the images of Fp or NADH reference standards. The images are labeled with numbers on the bottom and left axes to indicate the positions of the image matrices with a spatial resolution of 200 μm.

We have observed significant inter- and intratumor heterogeneity in the mitochondrial redox state in the breast cancer mouse xenografts. As the representative tumors shown in Figure 1, both aggressive lines (MDA-MB-231 and MDA-MB-468) displayed high degree of heterogeneity of NADH, Fp and the Fp redox ratio in each section, with localized areas (usually tumor central regions) exhibiting much higher Fp redox ratio than the other regions (usually tumor peripheries). A bimodal distribution is observed in their histograms. The indolent MCF-7 tumors (≤10 mm) were relatively less heterogeneous in each section, with a lower Fp redox ratio on average than that of the oxidized regions of the aggressive tumors. However, as we will show below, the MCF-7 tumors with larger size also showed high degree of heterogeneity with a clear center-periphery pattern.

**Figure 2:**
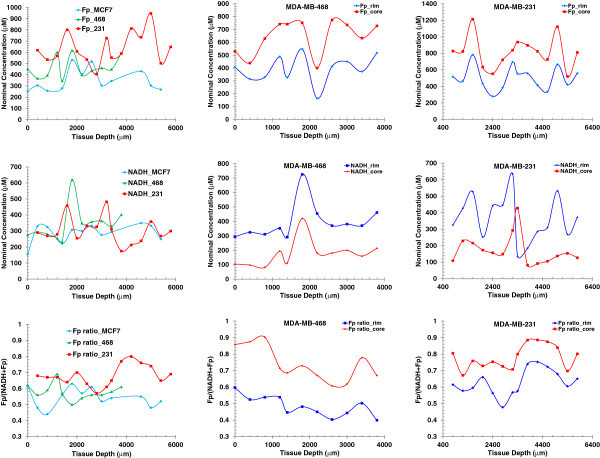
**Depth-dependence of redox indices (1**^**st**^**row: Fp; 2**^**nd**^**row: NADH; 3**^**nd**^**row: Fp redox ratio).** Left column: averaging over whole tumor sections for three lines; Middle and right column: the mean values in the core and rim of an MDA-MB-468 tumor and an MDA-MB-231 tumor, respectively.

To see how the redox indices change along the depth of each tissue section, they were plotted against the depth of each tumor section of the three tumors in Figure 1 to demonstrate how the redox indices distribute globally within a tumor (Figure 2). The left column of Figure 2 contains the plots of section average value of each of the redox indices versus depth. It is clearly seen that all three redox indices vary with depth for each tumor line. The aggressive tumors appear to have higher variations than the less aggressive MCF-7, demonstrating their higher degree of heterogeneity along section depth in addition to that exhibited on each section. We can see that the most aggressive tumor MDA-MB-231 has the highest Fp and the Fp redox ratio values, indicating it is the most oxidized tumor.

In addition to the section average values, for the aggressive tumors, we also obtained the mean values of the redox indices of the core (defined as high Fp redox ratio regions) and those of the rim (defined as low Fp redox ratio regions) by fitting of two Gaussian functions to their corresponding histograms. The plots in the middle column of Figure 2 are for the MDA-MB-468 tumor. We can see that the core and the rim are clearly separated for all three redox indices in the entire range of tissue depth. Fp is higher in the core area but drops sharply at 2200 μm and similar pattern is seen for Fp in the rim area. NADH distributions are roughly opposite to those of Fp with the maxima at 1900 μm. The Fp redox ratio is markedly higher in the core than in the rim. In comparison, the redox indices of the core and rim of the MDA-MB-231 tumor exhibited a different depth-variation pattern, but had a similar separation between the rim and core except at one depth point for NADH (Figure 2, right column). All these patterns of the aggressive tumors can be characterized by a bimodal distribution of the mitochondrial redox state with a more and a less oxidized region.

**Figure 3:**
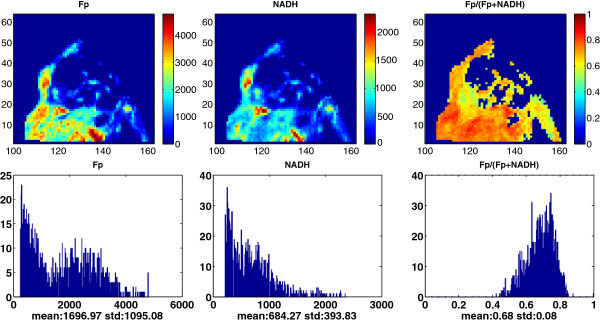
**Typical mitochondrial redox images and the corresponding histograms of breast tumor tissue from a patient.** Image matrix 256 × 64 (only partially displayed) and spatial resolution 100 μm.

Figure 3 displays the typical redox images of a breast cancer tissue block from a patient, who had an ER positive (>90%) invasive lobular carcinoma (ILC) and treated by arimidex for close to 8 months before her mastectomy. The heterogeneity is readily spotted from the images and also evidenced from the large standard deviations shown on the histograms. Heterogeneity of the redox indices was also evident from each of the core biopsy specimens of the cancerous tissue (Xu et al. 2013a).

### Simultaneous imaging of Pyro-2DG uptake and the redox state reveals islands of viable cells existed in the necrotic centers

**Figure 4:**
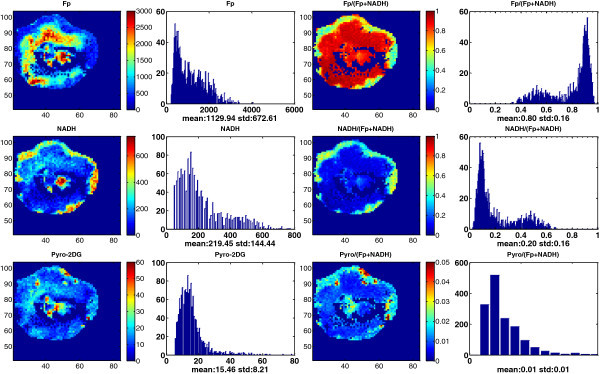
**Typical mitochondrial redox state and Pyro-2DG uptake images and their corresponding histograms of a representative section of a large MCF-7 tumor (>10 mm, Vol = 568 mm**^**3**^**).** The Fp redox ratio, NADH redox ratio, and normalized Pyro-2DG (Pyro-2DG/(Fp + NADH)) range between 0 and 1. The Fp, NADH, and Pyro-2DG images are in the unit of μM in reference to the corresponding standards. For other notations see the legend of Figure 1.

**Figure 5:**
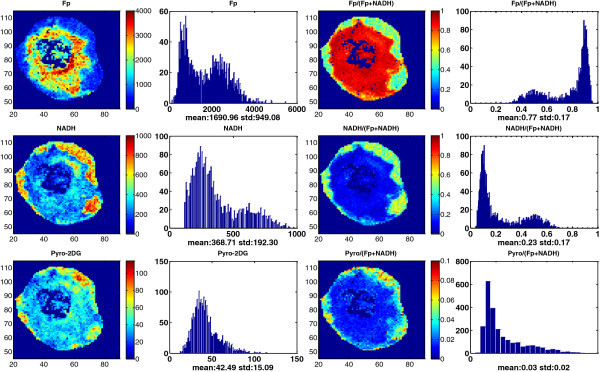
**Typical mitochondrial redox state and Pyro-2DG uptake images and their corresponding histograms of a representative section of a large MDA-MB-231 tumor (>10 mm, Vol = 766 mm**^**3**^**).** See the legend of Figure 4 for notations.

Figure 4 & 5 show the images of Fp, NADH, and pyro-2DG simultaneously acquired for two representative large and necrotic tumors of MCF-7 and MDA-MB-231, respectively. Also shown in these figures are the redox ratio images and those of pyro-2DG/(Fp + NADH), which are less sensitive to cell density variations by normalizing the concentrations to Fp + NADH. We again observed significant intra-tumor heterogeneity of the redox state and glucose uptake in both tumors, and their corresponding histograms also exhibited a bimodal or multi-modal distribution. Note that the large MCF-7 tumors had a core-rim pattern as the MDA-MB-231 tumors, which is not usually observed for smaller MCF-7 tumors (≤10 mm).

Due to complete cell death, we can identify certain central regions where there were no fluorescence signals in all channels. However, within these necrotic centers, there are islands of cells exhibiting active metabolism indicated by strong NADH, Fp, and Pyro-2DG signals, particularly in the large MCF-7 tumor in Figure 4. Two islands of viable cells in the tumor central region exhibit differential Pyro-2DG uptake as shown by the Pyro-2DG/(Fp + NADH) image although their corresponding redox ratios are similar.

**Figure 6:**
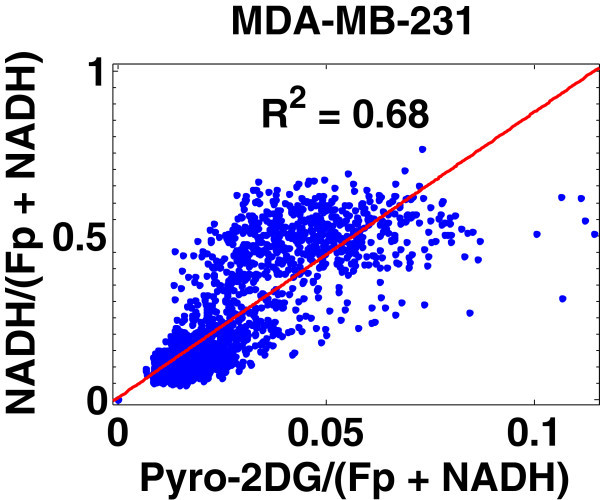
**Positive correlation between Pyro-2DG/(Fp + NADH) and NADH/(Fp + NADH) for the MDA-MB-231 tumor in Figure**5

In general, all four tumors had oxidized core and reduced peripheral regions mostly corresponding with the lower and the higher pyro-2DG uptake, respectively, and showed a positive correlation of the pyro-2DG signals with NADH or NADH redox ratios in peripheral tissue regions. Figure 6 is a plot of the distribution of the metabolic indices, i.e., the normalized glucose uptake Pyro-2DG/(Fp + NADH) versus the NADH redox ratio NADH/(Fp + NADH) for the MDA-MB-231 tumor image section displayed in Figure 5. A positive correlation is readily identified between these two indices and the linear regression fit yields a correlation coefficient R^2^ = 0.68. For the MCF-7 tumor image section in Figure 4, the positive correlation between Pyro-2DG/(Fp + NADH) and NADH/(Fp + NADH) is not as obvious and the linear regression fit gives R^2^ = 0.24. Similar correlations between Pyro-2DG or Pyro-2DG/(Fp + NADH) versus the redox indices (NADH/(Fp + NADH) or NADH) were observed in general for all four tumors. Despite the existing correlations, the distributions of the glucose uptake level versus mitochondrial NADH or NADH redox ratio were relatively scattered. On the correlation plots such as in Figure 6, for each normalized pyro-2DG uptake level, there are a number of pixels with different values of redox indices, and vice versa. Since the redox indices such as NADH and the NADH redox ratio are indicators of the mitochondrial redox state, these observations demonstrate that each pyro-2DG uptake level corresponds to a number of different mitochondrial redox states, and each mitochondrial redox state corresponds to a number of glucose uptake levels. This result indicates that there is a whole variety of cellular metabolic states existing in a breast tumor.

## Discussion

Tumor functional and metabolic heterogeneity has long been recognized. Studies have shown that clinical tumors exhibited more heterogeneous tissue oxygenation distributions than normal tissue, and the intertumor heterogeneity of tissue oxygenation was larger than intratumor heterogeneity; furthermore, the pO_2_ variations within breast tumors measured by needle electrodes did not correlate with the measuring site (tumor center versus periphery) (Vaupel et al. 2007; Vaupel et al. 1991). In addition to distinguishing between normal and (pre)malignant tissues (Xu et al. 2013b; Xu et al. 2013a; Xu et al. 2011b), imaging the intratumor heterogeneity of the mitochondrial redox state in tumors may provide useful information for understanding tumor aggressiveness (Li 2012). Our previous studies showed that more oxidized mitochondrial redox state in tumor tissue was positively associated with tumor metastatic potential in the melanoma and breast cancer xenograft models (Li et al. 2009a; Xu et al. 2010). We also observed that the clinical cancerous breast tissue was much more oxidized and more heterogeneous than the normal tissue (Xu et al. 2013a). The present study shows with great details of the metabolic heterogeneity in breast tumor xenografts and breast tumor specimens, which may be further investigated for developing high resolution biomarkers for clinical applications.

The present study is the first to reveal the 3D distribution of the mitochondrial redox state and Fp and NADH distribution in entire breast tumor xenografts. The three tumor lines we used in the study have the following ascending aggressiveness or invasive/metastatic potential, MCF-7 < MDA-MB-468 < MDA-MB-231 (Freund et al. 2003). Both MDA-MB-468 and MDA-MB-231 cells are triple-negative (negative for estrogen receptor, Her2/neu, and progesterone receptor) (Köster et al. 2009 ; Bartholomeusz et al. 2010; Anders & Carey 2008) and belong to aggressive tumors. From the redox images, we have observed significantly higher degree of heterogeneity in the more metastatic MDA-MB-231 and MDA-MB-468 tumors and less heterogeneity in the less metastatic MCF-7 tumors (≤10 mm). More quantitative analysis of the redox indices and their correlation with the aggressiveness of these three xenograft models is in progress. We also observed that MCF-7 tumors larger than 10 mm in diameter became more heterogeneous with distinct core-rim imaging pattern and the oxidized regions having very high Fp redox ratio (Figure 4). This result suggested that the large MCF-7 tumors may have acquired more aggressive features, consistent with the clinical observations that larger tumors tend to be more aggressive/metastatic.

We also investigated the glucose uptake by simultaneously imaging the fluorescence tracer pyro-2DG and the mitochondrial redox state in four large tumors. The results indicated that there were a large variety of cellular metabolic states existing under the complex tumor microenvironment. Although there are intertumor variations, the common patterns are that these tumors all exhibited more oxidized states in tumor central regions and more reduced states in tumor peripherals, and the normalized glucose uptake pyro-2DG/(Fp + NADH) grossly positively correlated with NADH/(Fp + NADH) in the tumor periphery. Early studies using bioluminescent enzymatic assay demonstrated the heterogeneous spatial distribution of glucose, lactate, and ATP in human breast cancer xenografts in rats (Kallinowski et al. 1988). Heterogeneous uptake of glucose by cancer cells in tumor xenografts was also quantitatively evaluated by radioluminography showing the selective uptake of deoxyglucose for viable cells over necrotic regions (Dearling et al. 2004). Such selectivity was also seen in the present study where tumor peripheral areas have apparently higher Pyro-2DG uptake, corresponding to higher NADH and the NADH redox ratio. This can be explained by the presumably higher perfusion and nutrient delivery and more NADH being metabolically generated in the tumor peripheral areas than in the central regions.

We also observed pronounced Pyro-2DG uptake in the areas inside the necrotic center along with significant NADH and Fp signals. High glucose uptake and NADH concentration inside the necrotic center indicate that these cells had high metabolic activities. It is likely that these cells were in State 3 where cells had sufficient nutrient resulting in rapid oxidative metabolism (high NADH, Fp and the Fp redox ratio) rather than in State 2 where cells were nutrient-starved (low NADH, high Fp and the Fp redox ratio) (Chance & Williams 1955 ; Chance & Baltscheffsky 1958; Chance & Schoener 1966). This also provides a possible answer to our previous question regarding whether the cancer cells in the oxidized regions were in State 2 (starvation) or State 3 (sufficient substrates) (Li et al. 2009a; Xu et al. 2010). According to the present study, it seems that the answer is some were in State 2 and some in State 3.

**Figure 7:**
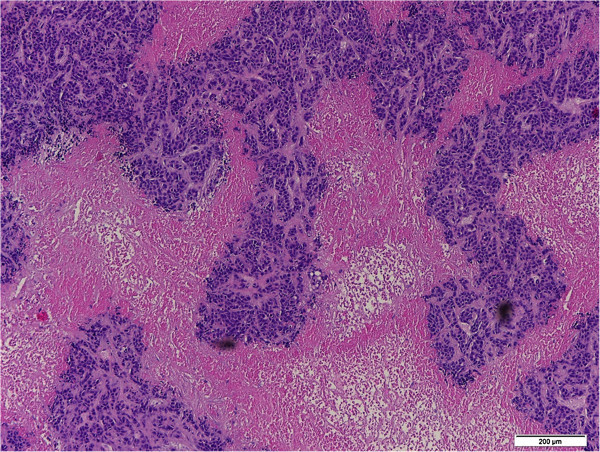
**Typical H&E staining of an area within the necrotic center of a large MCF-7 tumor (>10 mm) showing islands of viable cells (bar = 200 μm).**

The metabolic status of the cells in the necrotic center has not received noticeable attentions. The cells in the necrotic area are often considered dead due to limited blood perfusion that usually happens for aggressive tumors in mouse xenografts (Li et al. 2009a; Bradley et al. 2007; Pedley et al. 2001; Ranney et al. 2005). However, “hot” spots with high uptake of radiolabeled glucose were reported to exist in the necrotic areas of tumors in the literature (Dearling et al. 2004). Our previous studies on metastatic melanoma mouse xenografts also found that there was a sizable portion of cells inside the necrotic centers with intact nuclei shown by both the H&E staining and DAPI staining as well as TUNEL negativity, all of which indicated they were viable (Xu et al. 2010; Xu et al.2009b). The H&E staining of breast tumor xenografts also showed islands of cells with intact nuclei in the necrotic/oxidized regions (Figure 7). It is the redox indices in the oxidized tumor core that readily distinguish between the aggressive and indolent xenografts, better than the whole section average (Li et al. 2009a; Xu et al. 2010). These apparently viable cells in the oxidized cores may provide important clues to understanding tumor aggressiveness or metastatic potential.

## Conclusions

We reported the preliminary results from 3D high resolution mapping of the mitochondrial redox state of three breast cancer lines xenografted in mice, i.e., MCF-7, MDA-MB-468, and MDA-MB-231 with increasing ranks of metastatic potential. We identified significant intertumor and intratumor heterogeneity of the redox state among all xenografts and for the first time revealed the mitochondrial redox state spatial distribution in an entire tumor. Additionally, we showed that high degree of heterogeneity existed in the clinical biopsy samples from the breast cancer patients. We also reported the preliminary results of simultaneous imaging of the mitochondrial redox state and glucose uptake in larger breast cancer xenografts with apparent necrotic centers. We found that the glucose uptake was also heterogeneous (usually higher in tumor periphery) and positively correlates with NADH and the NADH redox ratio in general but not in all regions, and that islands of viable cells existed in tumor necrotic/oxidized centers exhibiting both high glucose uptake and NADH. These results indicated that a variety of metabolic states exist in a breast tumor and the metabolic heterogeneity warrants further investigation for developing potential metabolic biomarkers for clinical applications.
